# Granulocyte colony-stimulating factor affects the distribution and clonality of *TRGV *and *TRDV *repertoire of T cells and graft-versus-host disease

**DOI:** 10.1186/1479-5876-9-215

**Published:** 2011-12-15

**Authors:** Li Xuan, Xiuli Wu, Yu Zhang, Zhiping Fan, Yiwen Ling, Fen Huang, Fuhua Zhang, Xiao Zhai, Qifa Liu

**Affiliations:** 1 Department of Hematology, Nanfang Hospital, Southern Medical University, Guangzhou 510515, China

## Abstract

**Background:**

The immune modulatory effect of granulocyte colony-stimulating factor (G-CSF) on T cells resulted in an unexpected low incidence of graft-versus-host disease (GVHD) in allogeneic peripheral blood stem cell transplantation (allo-PBSCT). Recent data indicated that gamma delta^+ ^T cells might participate in mediating graft-versus-host disease (GVHD) and graft-versus-leukemia (GVL) effect after allogeneic hematopoietic stem cell transplantation. However, whether G-CSF could influence the T cell receptors (TCR) of gamma delta^+ ^T cells (*TRGV *and *TRDV *repertoire) remains unclear. To further characterize this feature, we compared the distribution and clonality of *TRGV *and *TRDV *repertoire of T cells before and after G-CSF mobilization and investigated the association between the changes of TCR repertoire and GVHD in patients undergoing G-CSF mobilized allo-PBSCT.

**Methods:**

The complementarity-determining region 3 (CDR3) sizes of three *TRGV *and eight *TRDV *subfamily genes were analyzed in peripheral blood mononuclear cells (PBMCs) from 20 donors before and after G-CSF mobilization, using RT-PCR and genescan technique. To determine the expression levels of *TRGV *subfamily genes, we performed quantitative analysis of *TRGV*I~III subfamilies by real-time PCR.

**Results:**

The expression levels of three *TRGV *subfamilies were significantly decreased after G-CSF mobilization (*P *= 0.015, 0.009 and 0.006, respectively). The pattern of *TRGV *subfamily expression levels was *TRGV*II >*TRGV *I >*TRGV *III before mobilization, and changed to *TRGV *I >*TRGV *II >*TRGV *III after G-CSF mobilization. The expression frequencies of *TRGV *and *TRDV *subfamilies changed at different levels after G-CSF mobilization. Most *TRGV *and *TRDV *subfamilies revealed polyclonality from pre-G-CSF-mobilized and G-CSF-mobilized samples. Oligoclonality was detected in *TRGV *and *TRDV *subfamilies in 3 donors before mobilization and in another 4 donors after G-CSF mobilization, distributed in *TRGV*II, *TRDV*1, *TRDV*3 and *TRDV*6, respectively. Signiﬁcant positive association was observed between the invariable clonality of *TRDV*1 gene repertoire after G-CSF mobilization and low incidence of GVHD in recipients (*P *= 0.015, *OR *= 0.047).

**Conclusions:**

G-CSF mobilization not only influences the distribution and expression levels of *TRGV *and *TRDV *repertoire, but also changes the clonality of gamma delta^+ ^T cells. This alteration of *TRGV *and *TRDV *repertoire might play a role in mediating GVHD in G-CSF mobilized allo-PBSCT.

## Background

Recently, the peripheral blood stem cells (PBSCs) obtained from granulocyte colony-stimulating factor (G-CSF) mobilized donors has been used more frequently than bone marrow stem cells as the source of stem cells in allogeneic hematopoietic stem cell transplantation (allo-HSCT). The clinical advantages of G-CSF-mobilized peripheral blood stem cell transplantation (G-PBSCT) accelerate engraftment and shorten the neutropenic period compared with bone marrow transplantation (BMT)[1,2]. In G-CSF mobilized allogeneic peripheral blood stem cell transplantation (allo-PBSCT), despite the presence of a more than 10-fold higher number of mature T cells in the graft, the incidence or severity of graft-versus-host disease (GVHD), especially acute GVHD, is not elevated compared with BMT [2,3]. Some studies suggested that the protective effects of G-CSF against GVHD might result from the immune modulatory effect of G-CSF on T cells, including that G-CSF directly modulated via its receptor on T cells or indirectly modulated T cell immune responses via effector cells and cytokines [4-8].

T cells recognize specific ligands by specific T cell receptors (TCR), which are heterodimers comprising either α/β or γ/δ chains. Genes encoding the variable domains of the TCR γ and δ heterodimer chains are *TRG *(γ chain) and *TRD *(δ chain), which are assembled by somatic recombination from variable (V), diversity (D, only for *TRD*), and joining (J) segments [9-11]. The *TRG *gene contains at least 14 functional variable (*TRGV*) segments belonging to four subgroups (*TRGV*I to IV), and the *TRD *contains at least 8 functional *TRDV *segments, which are subdivided into 8 *TRDV *subfamilies (*TRDV*1 to*TRDV*8)[12-14]. The functional capacities of γδ^+ ^T cells include cytokine production and potent cytotoxic effector activity [15-17]. Recently, it was reported that γδ^+ ^T cells might participate in regulation of autoimmune diseases and GVHD [16,18-21]. However, it is still unclear whether G-CSF mobilization could influence γδ^+ ^T cells and thereby mediate GVHD. In the present study, to further investigate the immune modulatory effect of G-CSF on T cells, we characterized the distribution and clonality of *TRGV *and *TRDV *subfamilies of donor T cells before and after G-CSF mobilization.

## Methods

### Samples

Peripheral blood was obtained from 20 healthy stem cell donors (9 female, 11 male; median age 30 years, range 14-56 years) before mobilization and on fifth day of mobilization with G-CSF (Filgrastim, subcutaneous injection of 5 μg/kg/d; Kirin Brewery Co, Tokyo, Japan). Peripheral blood mononuclear cells (PBMCs) were isolated from peripheral blood samples by Ficoll-Hypaque gradient centrifugation. The twenty healthy stem cell donors were willing to accept the trial after being informed, and all samples were obtained with consent from them. All the procedures were conducted according to the guidelines of the local ethical review boards before study initiation.

### RNA isolation and cDNA synthesis

RNA was extracted from the PBMCs of donors before and after G-CSF mobilization according to the manufacturer's protocol (Trizol, Invitrogen, USA). The quality of RNA was analyzed in 0.8% agarose gel stained with ethidium bromide. Two μg RNA was reversely transcribed into the first single-stranded cDNA with random hexamer primers, using reverse transcriptase of the Superscript II Kit (Gibco, USA). The quality of cDNA was confirmed by RT-PCR for β_2 _microglubin (β_2_M) gene amplification.

### RT-PCR for *TRGV *and *TRDV *subfamily amplification

As *TRGV*IV is a pseudogene [16], the analysis of *TRGV *repertoire was acquired in three *TRGV *subfamilies in the present study. Three sense *TRGV *primers and a single *TRGC *reverse primer, or 8 *TRDV *sense primers and a single *TRDC *primer were used in unlabeled PCR for amplification of the *TRGV *and *TRDV *subfamilies respectively. Subsequently, a runoff PCR was performed with fluorescent primers labeled at 5'end with the FAM fluorophore (Cγ-FAM or Cδ-FAM). Aliquots of the cDNA (1 μl) were amplified in 20 μl mixture with one of the 3 Vγ primers and a Cγ primer or one of 8 Vδ primers and a Cδ primer. The final mixture contained 0.5 μM sense primer and antisense primer, 0.1 mM dNTP, 1.5 mM MgCl_2_, 1 × PCR buffer and 1.25 U Taq polymerase (Promega, USA). The amplification was performed on a DNA thermal cycler (BioMetra, Germany) with 3 min denaturation at 94°C and 40 PCR cycles. Each cycle consisted of 94°C for 1 min, 60°C for 1 min and 72°C for 1 min respectively, and a final 7 min elongation at 72°C. All PCR products were stored at 4°C and ready for genescan analysis [16,22].

### Genescan analysis for *TRGV *and *TRDV *subfamily clonality

Aliquots of the unlabeled PCR products (2.5 μl) were separately added to a final 10 μl reaction system containing 0.1 μM Cγ-FAM or Cδ-FAM primer, 0.2 mM dNTP, 3 mM MgCl_2_, PCR buffer and 0.25 U Taq polymerase (Promega, USA). After a 3 min denaturation at 94°C, 35 cycles of amplification were carried out (1 min at 94°C, 1 min at 66°C and 1 min at 72°C and a final 6 min elongation at 72°C). The labeled runoff PCR products (2.5 μl) were heat denatured at 94°C for 4 min with 9.5 μl formamide (Hi-Di Formamide, ABI, USA) and 0.5 μl of Size Standards (GENESCAN™-500-LIZ™ Perkin Elmer, ABI). The samples were then loaded on 3100 POP-4™ gel (Performance Optimized Polymer-4, ABI, USA) and resolved by electrophoresis in 3100 DNA sequencer (ABI, Perkin Elmer) for size and fluorescance intensity analysis using Genescan software [16,22].

### Real-time quantitative PCR (RQ-PCR) for *TRGV *gene

Real-time PCR with SYBR Green I technique was used to examine *TRGV*I-III subfamily gene expression level in cDNA of PBMCs from 20 peripheral blood samples, the β_2_-microglobulin gene was used as an internal reference, the folds of change of *TRGV*I-III gene expression level were used by the 2^-ΔCt ^method. Briefly, PCR in 20 μl total volume was performed with approximately 1 μl cDNA, 0.5 μM of each primer (one of the three Vγ I-III sense primer and the antisense primer Cγ for *TRGV*I-III amplification, β_2_M-for and β_2_M-back primers for β_2_-microglobulin gene amplification) and 2.5 × RealMasterMix 9 μl (Tiangen, China). After the initial denaturation at 95°C for 2 min, 45 cycles consisting of 95°C 15 s, 60°C 60 s and 82°C 1 s for plate reading were performed using MJ Research DNA Engine Opticon 2 PCR cycler (BIO-RAD, USA). The relative mRNA expression level of *TRGV*I-III gene in each sample was calculated according to the comparative cycle time (Ct) method. Briefly, the target PCR Ct value, that is, the cycle number at which emitted fluorescence exceeds the 10 × SD of baseline emissions, is normalized by subtracting the β_2_M Ct value from the target PCR Ct value, which gives the ΔCt value. From this ΔCt value, the relative expression level to β_2_M for each target PCR can be calculated using the following equation: relative mRNA expression = 2^-ΔCt ^× 100% (ΔCt = Ct_(*TRGV*) _- Ct_(β2M)_) [16,22].

### Statistical analysis

Univariate analyses were performed using the Wilcoxon matched pair test to compare medians of *TRGV *or *TRDV *subfamilies between pre-G-CSF-mobilized and G-CSF-mobilized groups. McNemar's test was used for comparison of the expression frequencies of *TRGV *or *TRDV *subfamilies between two groups. Differences in mRNA expression of *TRGV*I-III between two groups were analyzed using the Wilcoxon matched pair test. Kruskal-Wallis Test was used for comparison of different gene expression levels from three *TRGV *subfamilies before or after G-CSF mobilization, and bonferroni correction was used for pairwise comparisons. Binary logistic regression analysis was used to estimate the association between GVHD in recipients and the alteration of each *TRGV *and *TRDV *repertoire after G-CSF mobilization. GVHD was considered as the dichotomous dependent variable and the changes of all *TRGV *and *TRDV *repertoires after G-CSF mobilization were considered as the covariates. *P *< 0.05 was considered as statistically significant (*P *< 0.0167 was considered as statistically significant in bonferroni correction).

## Results

### The expression pattern and levels of *TRGV *repertoire before and after G-CSF mobilization

**Figure 1:**
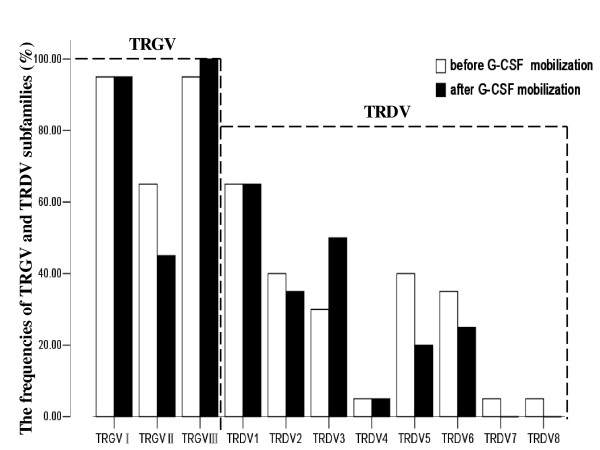
**The expression frequencies of *TRGV *and *TRDV *subfamilies in PBMCs from 20 donors before and after G-CSF mobilization**.

**Figure 2:**
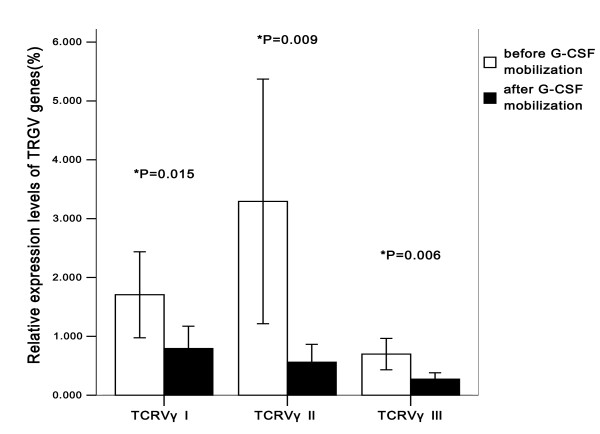
**The pattern of *TRGV *I-III expression levels in PBMCs from 20 donors before and after G-CSF mobilization**.

The CDR3 sizes and expression levels of three *TRGV *subfamily genes in T cells were respectively analyzed by RT-PCR in 20 donors before and after G-CSF mobilization. The results showed that the expression frequencies of *TRGV*I, *TRGV*II and *TRGV*III before mobilization were 95% (19/20), 65% (13/20) and 95% (19/20), respectively. After G-CSF mobilization, similar expression frequencies were found in *TRGV*I (95%, 19/20), *TRGV*II (45%, 9/20) and *TRGV*III (100%, 20/20) (P > 0.05) (Figure 1). Although the expression frequency of *TRGV*II decreased 20% after G-CSF mobilization, McNemar's test showed that there was no significant difference in expression frequency of *TRGV*II between pre-G-CSF and post-G-CSF group (*P *= 0.344). In addition, the expression levels of *TRGV*I, *TRGV*II and *TRGV*III genes after G-CSF mobilization were significantly lower than that before mobilization (*P *= 0.015, 0.009 and 0.006, respectively), quantified by 2^-ΔCt ^method (Figure 2). The pattern of *TRGV *expression levels before mobilization revealed as *TRGV*II >*TRGV*I >*TRGV*III, and there was a significant difference among the expression levels of three *TRGV *subgroups (*χ^2 ^*= 8.528, *P *= 0.014, Kruskal-Wallis test). However, after G-CSF mobilization, it revealed the *TRGV *I >*TRGV *II >*TRGV *III pattern and there was also a significant difference among three *TRGV *subfamily groups (*χ2 *= 6.933, *P *= 0.031, Kruskal-Wallis test) (Figure 2). Bonferroni correction was applied to further compare the difference in each group, there were significant differences between *TRGV*I and *TRGV*III before and after G-CSF mobilization (*P *= 0.004, 0.007, respectively), but there were no signiﬁcant difference between *TRGV*II and *TRGV*III (*P *= 0.031, 0.163, respectively), *TRGV*I and *TRGV*II (*P *= 0.582, 0.301, respectively) before and after G-CSF mobilization.

### The expression pattern of *TRDV *repertoire before and after G-CSF mobilization

In *TRDV *subfamilies, the number of detectable subfamilies ranged from 0 to 6 (median 2.12) before mobilization, which was higher than that after mobilization (ranged from 0 to 4, median 1.94, *P *> 0.05). The frequently expressed members were *TRDV*1 (65%, 13/20), *TRDV*2 and *TRDV*5 (40%, 8/20), *TRDV*6 (35%, 7/20) and *TRDV*3 (30%, 6/20) before G-CSF mobilization, while *TRDV*1 (65%, 13/20), *TRDV*3 (50%, 10/20), *TRDV*2 (35%, 7/20), *TRDV*6 (25%, 5/20) and *TRDV*5(20%, 4/20) after G-CSF mobilization. *TRDV*7 and *TRDV*8 were detected only in one case before mobilization, and were not identified in all samples after G-CSF mobilization (Figure 1). It could be seen from Figure 1 that the alteration in the expression frequencies between two groups was mainly embodied in *TRDV3 *(20%, 4/20) and *TRDV5 *(20%, 4/20), *TRDV6 *(10%, 2/20). However, there was no significant difference between pre-G-CSF and post-G-CSF expression frequencies of *TRDV*3, *TRDV*5 and *TRDV*6 (*P *= 0.344, *P *= 0.219, *P *= 0.688, respectively).

### The clonality of *TRGV *and *TRDV *subfamily T cells before and after G-CSF mobilization

**Figure 3:**
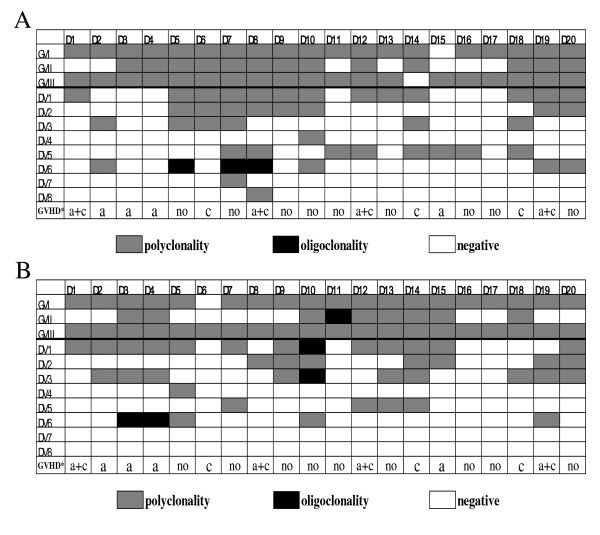
**Distribution and clonality of *TRGV *and *TRDV *subfamilies in PBMCs from 20 donors (D1-D20)**. (A) before G-CSF mobilization; (B) after G-CSF mobilization. GVHD*: the status of graft-versus-host disease of the corresponding recipients after allogeneic peripheral blood stem cell transplantation; a: acute graft-versus-host disease; c: chronic graft-versus-host disease.

**Figure 4:**
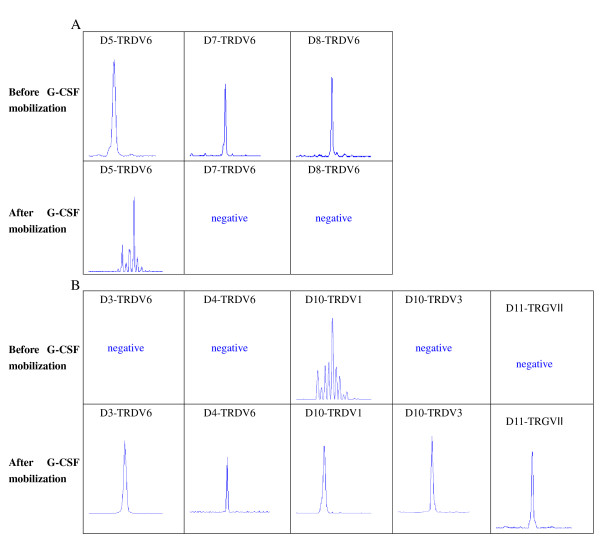
**Changes of clonality of *TRGV *and *TRDV *subfamilies in PBMCs from 7 donors (D5, D7, D8, D3, D4, D10 and D11) before and after G-CSF mobilization**. (A) Oligoclonal expansion changed to polyclonality or negative after mobilization; (B) Polyclonal or negative expansion changed to oligoclonality after mobilization.

To compare the differences in *TRGV *and *TRDV *gene repertoire diversity before and after G-CSF mobilization, three *TRGV *and eight *TRDV *gene transcripts and profiles were examined using genescan analysis. Most PCR products of *TRGV *and *TRDV *subfamilies displayed a Gaussian distribution of CDR3 lengths (multi-peaks) before and after G-CSF mobilization, which corresponded to polyclonal rearrangement pattern, whereas three PCR products in *TRDV*6 subfamily from 20 samples displayed oligoclonality before mobilization (Figure 3A). Clonal expansion was identified in another four cases after G-CSF mobilization, distributed in *TRGV*II, *TRDV*6, *TRDV*1 and *TRDV*3 (Figure 3B). The three oligoclonal expanded *TRDV*6 T cells which were identified before mobilization underwent alterations after G-CSF mobilization, in which two cases changed to negative and another one changed to polyclonality. Meanwhile, two cases with *TRDV*6 absence and one case with *TRGV*II absence before mobilization all changed to oligoclonality after G-CSF mobilization. Moreover, one case without *TRDV*3 expression changed to oligoclonality, and its polyclonal expansion in *TRDV*1 changed to oligoclonality after G-CSF mobilization (Figure 4). The alteration of clonality of *TRGV *and *TRDV *subfamilies between two groups was mainly reflected in *TRGV*II (50%, 10/20), *TRDV3 *(50%, 10/20), *TRDV1 *(45%, 9/20), *TRDV6 *(35%, 7/20)*, TRDV5 *(30%, 6/20), and *TRDV2 *(25%, 5/20) (Figure 3A and 3B).

### Outcome in recipients undergoing allo-PBSCT

From March 2010 to October 2010, 20 patients received G-CSF-mobilized allo-PBSCT from HLA-identical sibling donors. In total, 11 recipients experienced GVHD after transplantation, including 8 recipients with acute GVHD (grade I in 3 and gradeII in 5) and 7 recipients with chronic GVHD (3 extensive and 4 local). Thereinto, 4 cases with chronic GVHD experienced acute GVHD. We analyzed the association between GVHD and the alteration of clonality of each *TRGV *and *TRDV *repertoire after G-CSF mobilization. Simple effect analysis of binary logistic regression showed that the invariable clonality of *TRDV*1 gene repertoire after G-CSF mobilization was associated with low incidence of GVHD (*r *= 0.616, *P *= 0.004), and the alteration of other *TRDV *or *TRGV *subfamilies had no significant association with GVHD. Multivariate analysis also showed that the invariable clonality of *TRDV*1 gene repertoire after G-CSF mobilization indicated low incidence of GVHD (*P *= 0.015, odds ratio (*OR*) = 0.047, 95% confidence interval (*CI*): 0.004-0.552), and it was no signiﬁcant association between GVHD and the alteration of expression levels of three *TRGV *subgroups after G-CSF mobilization (*P *= 0.806, *P *= 0.458, *P *= 0.719, respectively). Meanwhile, it could be seen from Figure 3 that the incidence of GVHD was 8/11 (27.2%) in the *TRDV*1 clonality invariable group, whereas the incidence of GVHD reached 8/9 (88.9%) in the *TRDV*1 clonality variable group. In addition, the alteration of expression pattern of *TRGV *repertoire also was not significantly associated with GVHD (*P *= 0.120).

## Discussion

The immune modulatory effect of G-CSF on T cells resulted in an unexpected low incidence of GVHD in G-CSF mobilized allo-PBSCT. However, the underlying mechanism for the reduced reactivity or alloreactivity of T cells after G-CSF mobilization was not fully understood [23,24]. A growing body of experimental evidence suggests that G-CSF might interact with the immune system by altering T cell reactivity [23,25]. Some studies suggested that G-CSF could directly modulate T-cell immune responses via its receptor, which could be detected on T cells [24,26]. However, whether the unactivated T cells could express G-CSF receptor (G-CSFR) remains unclear. Franzke's study showed that the expression of G-CSFR was undetected in the unactivated T cells, but both CD4^+ ^and CD8^+ ^T cell could express the G-CSFR at the mRNA level after G-CSF stimulation in vivo or vitro [24]. Morikawa et al. also observed that the unactivated T cells from peripheral blood did not bind to the biotinylated G-CSF, but the active T cells after Con A stimulation could bind to the biotinylated G-CSF [26]. These studies suggested that the unactivated T cells could express G-CSFR by stimulation [23,24,26]. However, whether G-CSF directly modulates T-cell immune responses via G-CSFR on T cells or T cell receptor needs further investigation.

T cells are comprised of two major subpopulations, identiﬁed by their expression of either the αβ or γδ TCR heterodimer. αβ^+ ^T cells are the predominant circulating population and can be subdivided into cells that express CD4^+ ^or CD8^+ ^antigens. γδ^+ ^T cells, which represent approximately 5-10% of peripheral T cells and are predominantly CD3^+ ^CD4^- ^CD8^- ^T cells [16,27], recognize specific antigen without MHC-restriction and are considered as linkage between innate and adaptive immune response [28,29]. As a group of innate immune cells, γδ^+ ^T cells respond rapidly and expand efficiently when stimulated by antigens or cytokines and may be a good target for modulation of immune responses in human diseases. Recently, the role of γδ^+ ^T cells in mediating GVHD raised certain attention [30-36], but whether γδ^+ ^T cells promoted or inhibited the occurrence of GVHD was still controversial. Several studies showed that γδ^+ ^T cells might contribute to GVHD [32-34], while others demonstrated that γδ^+ ^T cells could inhibit GVHD [35,36]. The generation and maintenance of a diverse T-cell repertoire is a critical element in immune competence. Therefore, it might be interesting to further clarify whether G-CSF could influence the distribution and clonality of *TRGV *and *TRDV *repertoire of γδ^+ ^T cells, thereby influencing the alloreactivity of T cells and mediating GVHD in G-CSF mobilized allo-PBSCT.

In the present study, we investigated the feature of distribution and clonality of *TRGV *and *TRDV *repertoire from healthy donors before and after G-CSF mobilization. The results showed that G-CSF mobilization could influence the distribution and clonality of *TRGV *and *TRDV *repertoire of γδ^+ ^T cells. The expression levels of three *TRGV *subfamilies were significantly decreased after G-CSF mobilization. In addition, the pattern of *TRGV *expression levels also changed after G-CSF mobilization, suggesting that G-CSF might influence the expression pattern of *TRGV *subfamilies. The expression frequencies of *TRGV *and *TRDV *subfamilies changed at different levels after G-CSF mobilization; however, there were no significant differences in the expression frequencies of all *TRGV *and *TRDV *subfamilies between pre-G-CSF and post-G-CSF group. Most *TRGV *and *TRDV *subfamilies revealed polyclonality from pre-G-CSF-mobilized and G-CSF-mobilized samples. Oligoclonality was detected in *TRGV *and *TRDV *subfamilies in 3 donors before mobilization and in another 4 donors after G-CSF mobilization, distributed in *TRGV*II, *TRDV*1, *TRDV*3 and *TRDV*6, respectively. Recent studies suggested that some clonal T cells might be a reaction to host alloantigen and related with GVHD activity [37]. Meanwhile, it is recognized that the persistent oligoclonal T cell expansion could be incurred due to many factors affecting the overall regulation of clone size in response to chronic antigens [16,38]. Our studies revealed that the two oligoclonal expanded *TRDV*6 T cells appeared in two patients with aGVHD, while the expansion of the *TRGV*II T cells, as well as the expansion of the *TRDV*1 and *TRDV*3 T cells appeared in two patients without GVHD. Consequently, whether the change of clonality was due to the effect of G-CSF or it was related with GVHD needs further study. However, it was a pity that the donors were not followed up, as it took a long time to get the results.

Recently, researchers revealed that γδ^+ ^T cells produced a series of cytokines in pathology and played an indispensable role in pathogen elimination, immune regulation and autoimmunity [39,40]. γδ^+ ^T cells modulated immune responses mainly by secreting cytokines and by regulating the function of other immune cells, such as αβ^+ ^T cells, macrophages, NK cells and CD4^+^CD25^+^Foxp3^+ ^regulatory T cells (Treg)and so on [41-44]. Despite the variety of characteristics attributed to γδ^+ ^T cells, their exact role in mediating GVHD remained unclear. Drobyski et al. postulated that transplantation with γδ^+ ^T cells protected mice from GVHD [36], whereas Blazar et al. showed that the infusion of donor γδ^+ ^T cells induced lethal GVHD in mice [34]. However, Anderson et al did not find any correlation between host γδ^+ ^T cells and GVHD in mice [45]. Similarly in human studies, Pabst et.al showed that it was a significant association between an increased donor γδ^+ ^T cells dose and the cumulative incidence estimates of aGVHD [46], whereas Godder et al. observed that patients with increased γδ^+ ^T cells recovery did not have an increased incidence of GVHD compared to those with normal or decreased numbers [20].

Different from most studies which analyzed from angle of quantity of γδ^+ ^T cells, we studied from the perspective of different repertoire of γδ^+ ^T cells (*TRGV *and *TRDV *repertoire), which was thought to have different functions for immune response. Positive association was observed between the invariable clonality of *TRDV*1 gene repertoire after G-CSF mobilization and low incidence of GVHD, and the invariable clonality of *TRDV*1 gene repertoire after G-CSF mobilization indicated low incidence of GVHD (*OR *= 0.047). Due to the small sample size, the association between GVHD and other *TRDV *and *TRGV *subfamilies needs further study. These results suggested that compared with other *TRDV *and *TRGV *repertoire, some donors' *TRDV*1 repertoire might be more sensitive to GVHD-associated antigens after G-CSF mobilization, causing the occurrence of GVHD; whereas *TRDV*1 repertoire of most donors maintained stability after G-CSF mobilization, resulting in low incidence of GVHD. Therefore, *TRDV*1 repertoire might be used as an observation index for GVHD in the future. In short, our studies observed that some repertoire of γδ^+ ^T cells changed after G-CSF mobilization. Accordingly, the antigens recognized by them might change, thereby altering the immune responses of γδ^+ ^T cells and mediating the occurrence of GVHD. However, the mechanisms are still not well understood and need further study.

## Conclusions

We characterized the distribution and clonality of *TRGV *and *TRDV *subfamilies of donor T cells before and after G-CSF mobilization. The results show that G-CSF mobilization not only influences the distribution and expression levels of *TRGV *and *TRDV *repertoire, but also changes the clonality of γδ^+ ^T cells. This alteration of *TRGV *and *TRDV *repertoire might play a role in mediating GVHD in G-CSF mobilized allo-PBSCT.

## Competing interests

The authors declare that they have no competing interests.

## Authors' contributions

LX and XLW performed research, analyzed data and wrote the paper; YZ, ZPF and FH analyzed data; YWL, FHZ and XZ performed research; QFL designed research and wrote the paper. The authors reported no potential conflicts of interest. All authors read and approved the final manuscript.
